# Trends in Aquatic Environmental DNA Research in Alaska

**DOI:** 10.1002/ece3.73925

**Published:** 2026-06-30

**Authors:** Brandi Kamermans, Maggie Harings, Rachel Lekanoff, Laura E. Timm, Erik Schoen, J. Andrés López, Jessica R. Glass

**Affiliations:** ^1^ International Arctic Research Center University of Alaska Fairbanks Fairbanks Alaska USA; ^2^ College of Fisheries and Ocean Sciences University of Alaska Fairbanks Fairbanks Alaska USA; ^3^ National Marine Fisheries Service, Alaska Fisheries Science Center, Resource Ecology and Fisheries Management National Oceanic and Atmospheric Administration Seattle Washington USA; ^4^ University of Alaska Museum of the North, University of Alaska Fairbanks Fairbanks Alaska USA

**Keywords:** Arctic, biodiversity, biomonitoring, coproduction, management, subarctic

## Abstract

Environmental DNA (eDNA) analysis is an emerging tool with significant potential to advance biomonitoring, particularly in remote and logistically challenging environments. The state of Alaska, USA, contains such environments that create unique issues regarding access and sampling. To evaluate the state of eDNA research in Alaska, we conducted a literature review and a regional survey. The review identified 22 peer‐reviewed studies published between 2010 and 2025, while the survey of responses representing state, federal, academic, tribal, and nonprofit organizations (46 responses) captured information on ongoing and unpublished projects. Our literature review and survey results reveal that most published and ongoing studies in Alaska employ eDNA metabarcoding to assess community assemblages, species distributions, and biodiversity patterns. However, respondents reported several barriers to implementation, including limited funding, infrastructure, and assay availability. Barriers include uncertainty in laboratory selection, sampling protocols, and data analysis. Despite these challenges, cross‐sector collaborations are developing. Across the growing effort to harness eDNA as a management tool, collaborations with subsistence harvesters are emerging as a promising approach for sample collection in remote areas. This study provides the first comprehensive overview of eDNA research in Alaska, identifies key data gaps, and offers examples of coproduction of knowledge currently underway in the state. eDNA research strategies that address these data gaps and ongoing coproduction frameworks developed in Alaska inform and advance remote and Arctic biomonitoring programs globally.

## Graphical abstract

Environmental DNA (eDNA) analysis is an emerging tool with significant potential to advance biomonitoring, particularly in remote and logistically challenging environments. To evaluate the state of eDNA research in Alaska, we conducted a literature review and a regional survey and results provide the first comprehensive overview of eDNA research in Alaska, identify key data gaps, and offers examples of coproduction of knowledge currently underway in the state.

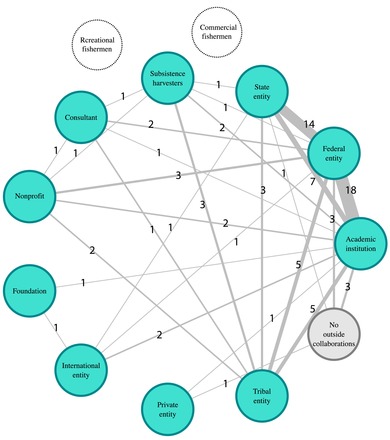

## 1 Introduction

Small‐ and large‐scale drivers, like shifts in temperature and transformations in land‐use, are affecting biodiversity worldwide (Genet et al. 2018; Rasmus et al. 2024; Wägele et al. 2022). Assessments of species distribution, abundance, and biodiversity are pivotal to understanding aquatic ecosystems, but aquatic biomonitoring programs are frequently hampered by limitations of scale, throughput, and cost (Carrizo et al. 2017; Dolman et al. 2012; Johnston et al. 2023). These limitations are particularly acute in remote regions, including high‐latitude and oceanic systems, where difficulty of access, inclement weather, and scarce laboratory facilities pose additional challenges (Cardenas et al. 2024; Chen et al. 2022; Reddy 2021; Wang et al. 2024). Environmental DNA (eDNA) is emerging as a powerful and cost‐effective tool for assessing and monitoring biodiversity across diverse environments, including aquatic ecosystems (Blackman et al. 2024; Deiner et al. 2017; Rourke et al. 2022).

The opportunities and challenges of applying eDNA approaches in remote aquatic environments are exemplified by an expanding body of research in Alaska. It is the largest, most sparsely populated and northernmost US state. Alaska is home to some of the most valuable fisheries in the world and boasts an extensive 66,640 miles (107,247 km) of coastline (Beaudreau et al. 2019) and approximately 3 million lakes, 12,000 rivers, and 34,000 miles of tidal shoreline (Milner and Oswood 1997). Researchers in Alaska have already demonstrated that eDNA methods can complement traditional monitoring approaches like weir counts (Levi et al. 2019) and net sampling (Deeg et al. 2023) when appropriately calibrated for environmental factors such as stream flow and DNA degradation (Levi et al. 2019; Matter et al. 2018; Pochardt et al. 2020). Multiple collaborations with agencies and subsistence harvesters are leveraged for monitoring programs. For instance, eDNA monitoring has been proven to be a more cost‐effective and less invasive option for long‐term monitoring of a culturally important fish species than a conventional mark‐recapture approach using net sampling (Pochardt et al. 2020). Many other applications of eDNA technology may be possible, especially if collaborations with agencies and subsistence harvesters are leveraged for monitoring programs.

With purportedly increased applications of eDNA in remote regions like Alaska, we identified the need for a synthesis article to determine the: (1) spatial and temporal trends and full breadth of organisms currently being studied, (2) variability in protocols for both field and laboratory methods, and (3) barriers faced by researchers. When examining how eDNA methodologies in Alaska have been interpreted and represented in the published literature, we found that existing reviews often overlooked Alaska‐based and Arctic‐specific approaches (Rees et al. 2014; Takahashi et al. 2023; Zhang et al. 2023; Capurso et al. 2023; Rishan et al. 2024; Sahu et al. 2025; Çevik and Çevik 2025; Stein et al. 2024; Wu et al. 2026; Iacaruso et al. 2026). The absence of Arctic perspectives perpetuates a bias toward more accessible, temperate systems. This gap also reflects a broader failure to integrate research from Indigenous managed landscapes. The Alaska‐ and Arctic‐specific approach are essential for advancing eDNA as a tool for natural resource management in understudied habitats critical to global biodiversity during rapid changes associated with climate change.

Fieldwork in Alaska is shaped by logistical constraints that are uncommon elsewhere and directly influence eDNA study design and implementation. For example, 80% of communities are not connected by road systems and access to sampling sites often requires aircraft, snowmachine, the use of seasonal routes (e.g., ice roads), or extended river travel, substantially increasing costs and limiting sampling opportunities. Researchers frequently rely on specialized skills such as operating snowmachines and boats. The sampling events must contend with extreme weather that can disrupt sampling schedules. Remote field conditions also introduce challenges related to equipment reliability, limited‐to‐no communication infrastructure, and lack of nearby laboratories for sample processing. In some cases, deploying or maintaining sampling infrastructure requires additional technical expertise (e.g., wiring autonomous systems to power sources).

While these factors are particularly pronounced in Alaska, they are broadly relevant to other remote or data‐limited regions, where similar constraints may shape eDNA workflows and contribute to variability in sampling approaches. Here, we review and synthesize the rapidly growing applications of eDNA analysis for ecosystem monitoring in the freshwater and marine environments of Alaska. This article aims to highlight overlooked methodologies developed for some of the most logistically challenging and ecologically sensitive environments.

## 2 Materials and Methods

### 2.1 Literature Review and Survey Development and Distribution

To characterize previous and ongoing eDNA methods used in aquatic ecosystems in Alaska, we conducted a literature review in November 2023 and updated it in December 2025. Scientific articles and reviews of aquatic eDNA science in Alaska were retrieved from Google Scholar, PubMed, JStor, Research Gate, Semantic Scholar (Table A1). We used the following words to find relevant papers: Arctic, Alaska, environmental, DNA, quantitative PCR, qPCR, metagenomics, Taqman, digital droplet PCR, ddPCR, digital PCR, dPCR, metabarcoding, primers, high‐throughput, illumina, sequence, biodiversity, monitoring, invasive, endangered, salmon (Table A1). To complement the review, we designed a survey to document published and unpublished research. We designed survey questions focused on the following: (1) the spatial and temporal trends and full breadth of organisms currently being studied, (2) variability in protocols for both field and laboratory methods, and (3) barriers faced by researchers. Survey questions surrounding key themes of study timeline, study regions, methods, ecosystems, and target species were included because they aligned with questions often asked by similar studies on a global scale (Capurso et al. 2023; Stein et al. 2024). Aligning our survey questions with prior published studies (Schenekar 2023; Takahashi et al. 2023) allowed us to interpret Alaska‐based research within the context of global trends. In addition, we included questions on collaborations and funding sources to shed light on the solutions researchers were finding for barriers. While these topics are less frequently addressed in comparable studies, our research team identified them as critical for understanding how eDNA research can be more strategically advanced in Alaska. The survey captured perspectives on perceived barriers to conducting eDNA research in Alaska (see survey in the Appendix). The survey was distributed digitally as a Google form and administered to the following: (1) individuals, (2) organizations (e.g., Tyonek Tribal Conservation District and the Kenai Watershed Forum), and (3) working group listservs (e.g., Alaska Invasive Species Partnership and the eDNA Collaborative). Individuals included eDNA researchers identified via personal communication, members of informal Alaska and Washington eDNA working group meetings, and researchers listed as authors on abstracts at conferences (American Fisheries Society, American Geophysical Union, Alaska Marine Science Symposium, and the 2nd National Workshop on Marine eDNA). Survey participants who chose to self‐identify are listed in the Acknowledgments.

### 2.2 Data Encoding

Raw survey responses and data from publications were compiled for analyses. For multiple choice questions and short answers, each response from each participant was treated as a separate data point. For example, respondents reported testing several different filter pore sizes during a single research project. Each unique size was considered a separate observation during data analysis. If a participant's response to a question was ambiguous, we categorized their answer as “unknown.”

Study regions included Bering Sea, Chukchi Sea, Beaufort Sea, Gulf of Alaska, Interior and North‐northwest, Southcentral, Southeast, Statewide, West‐southwest, and Aleutian Islands. In addition to finding locations in the published literature, the survey included a question asking the latitude and longitude of studies.

Funding sources were categorized as federal grant funding, state grant funding, Tribal grant funding, private funding, and/or other funding sources. Aquatic ecosystems sampled included ocean, lake, wetland, river/stream, tidewater, and/or estuary.

The survey included the following options for participants to choose from: fish (including lampreys), crustaceans, reptiles, mammals (marine), mammals (land‐based), mollusks, macroinvertebrates, plants (macrophytes), microalgae, and “other.” Survey responses identified several additional taxa, and some responses selected all taxa.

Filter pore sizes in microns (μm) included 0.1, 0.22, 0.4, 0.45–1.5, 1, 1.2, 1.5, 3, 5, 7, 10, unknown, and none (e.g., no filter was used and gauze and ethanol precipitation methods were utilized for capturing and concentrating eDNA in the field). Filter types included unknown, none (ethanol precipitation), glass microfiber (GMF), sterile gauze, polyethersulfone (PES), cellulose acetate, nylon, polycarbonate (PC), nylon net filter, and cellulose nitrate (CN). Contamination control implementation step(s) at which a sample blank was used included sample collection, filter subsampling, DNA extraction, DNA sequencing, other, or none.

Project types included presence/non‐detection, species quantification, rare species assessment, invasive species detection, eDNA ecology, field‐based method‐comparison, laboratory‐based method‐comparison, and/or assay development/validation. Rare species assessments and invasive species detection are special cases of presence/non‐detection, and we considered them separate types to capture. Participants could select both “presence/non‐detection” and “rare species assessment.” These definitions were chosen based on the eDNA literature. For example, presence/non‐detection studies use eDNA to test for the presence or absence of an organism (Perl et al. 2022) and species quantification studies use eDNA to measure species abundance (Baker et al. 2018; Wang et al. 2021; Yates et al. 2021). Presence/non‐detection and species quantification studies can target rare organisms (Carim et al. 2016; McKelvey et al. 2016; Wilcox et al. 2016) and detrimental non‐native species (Morisette et al. 2021). Metabarcoding is a type of presence/non‐detection that provides community‐level data that informs biodiversity and population assessments (Kelly, Port, Yamahara, Martone, et al. 2014; Kelly, Port, Yamahara, and Crowder 2014; Thomsen and Willerslev 2015). eDNA ecology studies focus on the range of biotic and abiotic environmental factors that contribute to eDNA persistence and the limits of detection (Barnes and Turner 2016; Dejean et al. 2011; Hansen et al. 2018). Field‐based method‐comparisons compare different environmental factors and sampling techniques on eDNA results (Larson et al. 2022; Matter et al. 2018), while laboratory‐based method‐comparison studies compare detection of eDNA with other sampling methods and other biodiversity measures (Dunker et al. 2016). Assay development/validation studies develop and assess new assays for species‐specific detection (Thalinger et al. 2021).

Collaborators included state agency, federal agency, tribal entity, academic institution, nonprofit, consultant, subsistence harvesters, recreational fishermen, commercial fishermen, and/or other.

Barriers included insufficient funding, lack of agency/organization support, lack of laboratory access/lack of funding for sample analyses, uncertainty about how to analyze data, “None” and/or “other” were also options. When a response to this question could not be parsed from literature, we recorded “unknown.”

Publication status included completed, published or ongoing, unpublished, and other. Seven publications became available after the survey closed in January 2024. Although they are included here for completeness, they were excluded from analyses and the figures. The analyses presented in this article represent the state of eDNA research in Alaska based on our literature review and regional survey to gather perspectives on barriers and collaborations. Some of the authors of these studies participated in the survey and indicated that their work was in progress or unpublished at the time. As such, including these studies in our analyses after the survey closed could result in duplication of survey responses. However, we aim to provide the readers with a complete list of publications which are summarized in Table A2.

### 2.3 Data Analysis

When summarizing the results of the multiple‐choice questions, we treated each answer as a unique entry. Therefore, if participants selected more than one answer (e.g., they were targeting two unique taxa or were affiliated with two types of institutions), the total number of entries could exceed the number of participants. In several cases, short‐answer responses provided under “other” required category revision, including grouping options and/or creating new options. Data categories are detailed below.

#### 2.3.1 Study Region

Binning was based on commonalities in geophysical location. The categories were broken up into: Bering/Chukchi Sea and Arctic Ocean (Bering, Chukchi, and Beaufort Sea), Gulf of Alaska, North of the Alaska Range (Interior and North‐northwest), Southcentral, Southeast, Statewide, and/or West‐Southwest (West‐southwest and Aleutian Islands).

#### 2.3.2 Targeted Taxa

For data analysis, we categorized the target taxa as: fishes, invertebrates (macroinvertebrates, crustaceans, mollusks, and cephalopods), mammals, multiple, pathogens, plants, and microalgae. These categories are based on previous reviews (Schenekar 2023; Takahashi et al. 2023).

#### 2.3.3 Funding

Binning was based on categories we included in the survey: federal, state, tribal, and/or other (international, private, pursuing funding, and unknown).

#### 2.3.4 Aquatic Ecosystems Sampled

Binning was based on categories we included in the survey: ocean, lentic systems (lake and wetland), lotic systems (river/stream), and/or coastal aquatic ecosystems (tidewater and estuary).

#### 2.3.5 Barriers

We asked participants to identify any barriers to performing eDNA research and provided the following options: (1) insufficient funding, (2) lack of agency/organization support, (3) lack of laboratory access/lack of funding for sample analyses, (4) uncertainty about how to analyze data, (5) None—I am well‐versed in eDNA methods and applications, and (6) “other.” Binning was based on commonalities among categories we included in the survey and barriers provided in “other” by survey participants. For example, uncertainty and skepticism were binned together because we provided the response, “uncertainty about how to analyze data” and barriers provided by respondents in “other” included the following: uncertainty about lab selection, skepticism from end‐users, and inconclusive results. These three additional barriers fit within the theme of uncertainty rather than other categories. Similarly, we binned responses to “lack of laboratory access/lack of funding for sample analyses,” and “lack of agency/organization support,” along with “other” responses, into a category called “lack of access to resources,” which referenced the following: remote sampling locations, facility infrastructure, sampling equipment, laboratory equipment, agency/organization support, existing assays specific to Alaskan species or regions, personnel trained in field and laboratory techniques, and organizational laboratory capacity.

#### 2.3.6 Filter Pore Size

Survey responses were collected as short answers and identified a range of filter pore sizes used, as well as categories for unknown and none.

#### 2.3.7 Filter Type

Participants added options such as “NA (ethanol precipitation),” “nylon,” and “nylon net filter” in the survey. We categorized filter types into the following groups: none (ethanol precipitation), nylon, nylon net filter, glass microfiber (GMF), sterile gauze, polyethylene sulfone (PES), cellulose acetate, polycarbonate (PC), and cellulose nitrate (CN).

#### 2.3.8 Negative Control Use

The use of negative controls within each study workflow was assessed by separating the workflow into the following: sample collection, filter subsetting, DNA extractions, DNA analyses (plate blanks used for qPCR and/or metabarcoding analyses). Some survey participants chose none and/or unknown for these steps.

#### 2.3.9 Collaborations

Participants chose from state agency, federal agency, tribal entity, academic institution, nonprofit, consultant, subsistence harvesters, recreational fishermen, commercial fishermen, and other.

### 2.4 Graphical Representation

We compiled and analyzed data survey responses and the information parsed from the literature review in RStudio (R version 4.3.2) (R Core Team (2024)). We generated figures and tables using R packages ggplot2 (Wickham 2016) and iGraph (Csárdi et al. 2024). To understand the historical use of eDNA in the state, we focused on eDNA analyses over space and time. Then, given the diverse interests in aquatic biomonitoring in Alaska, we sought to characterize studies by targeted taxa, aquatic ecosystems sampled, and publication status. We mapped the locations of studies across Alaska, including surrounding waters, distinguished by regional boundaries (e.g., Southcentral, North‐Northwest, Gulf of Alaska) that are used for natural resource management. We used the reported latitude and longitude from surveys and published studies to guide our understanding of where research has been conducted and for specific target groups of organisms. Moreover, to understand research interests across these regional boundaries and identify project funding sources (e.g., federal, state), we assessed the funding origins for projects in each region. Respondents to a recent survey (Capurso et al. 2023) suggested that better collaboration and sharing of knowledge and data among managers could improve monitoring strategies in the future; here, we also investigate the interconnectedness of the Alaska eDNA research teams.

## 3 Results

**FIGURE 1:**
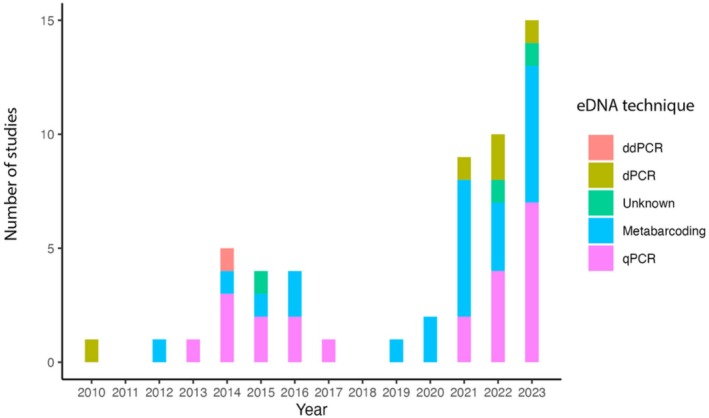
The stacked plot displays the number of combined unpublished and published eDNA studies initiated in 2010–2023. The studies are plotted based on the project start date. We examined the number of projects in the dataset by each year they were initiated and by project type.

Our literature review targeted 15 relevant peer‐reviewed articles on aquatic eDNA research in Alaska and adjacent marine ecosystems (Figure 1 and Tables A3 and A4). The application of eDNA in Alaska to monitor species has increased since 2010, with a more rapid increase since 2019 (Figure 1). Of authors corresponding to the 15 published studies in Alaska (*n* = 54), 85% (*n* = 46) participated in the survey.

**FIGURE 2:**
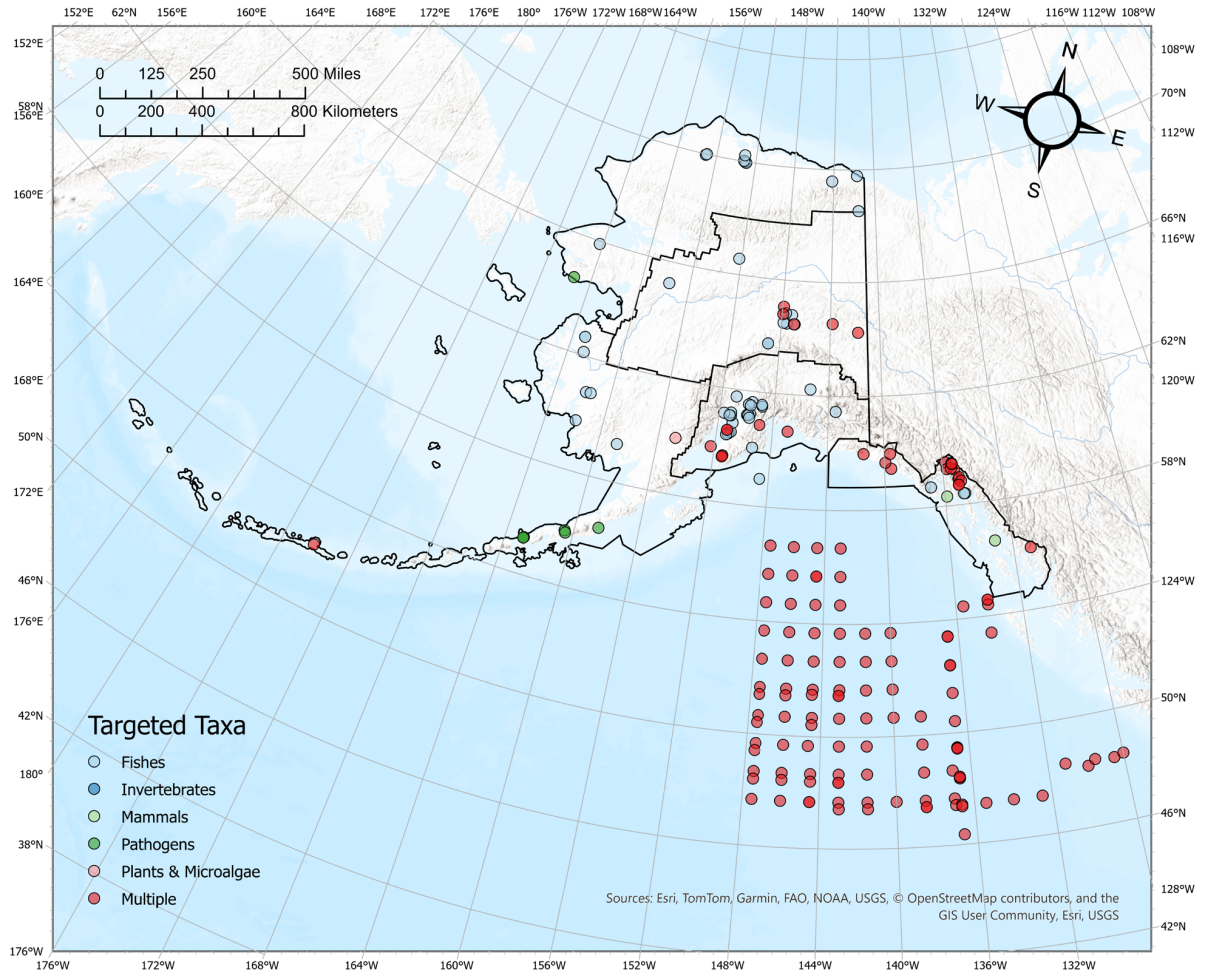
Map of Alaska with the location of eDNA projects that were initiated between 2010 and 2023. The colors represent the taxa targeted in each study type. Regional boundaries are outlined in black, except for the Gulf of Alaska (GOA; Northern Gulf of Alaska LTER [long‐term ecological research]). Samples collected in the Bering, Chukchi, and Beaufort Seas are colocated with long‐term EcoFOCI moored sites and the established Distributed Biological Observatories and are not shown on this map as these locations were not in the published report (Galaska et al. 2023).

**FIGURE 3:**
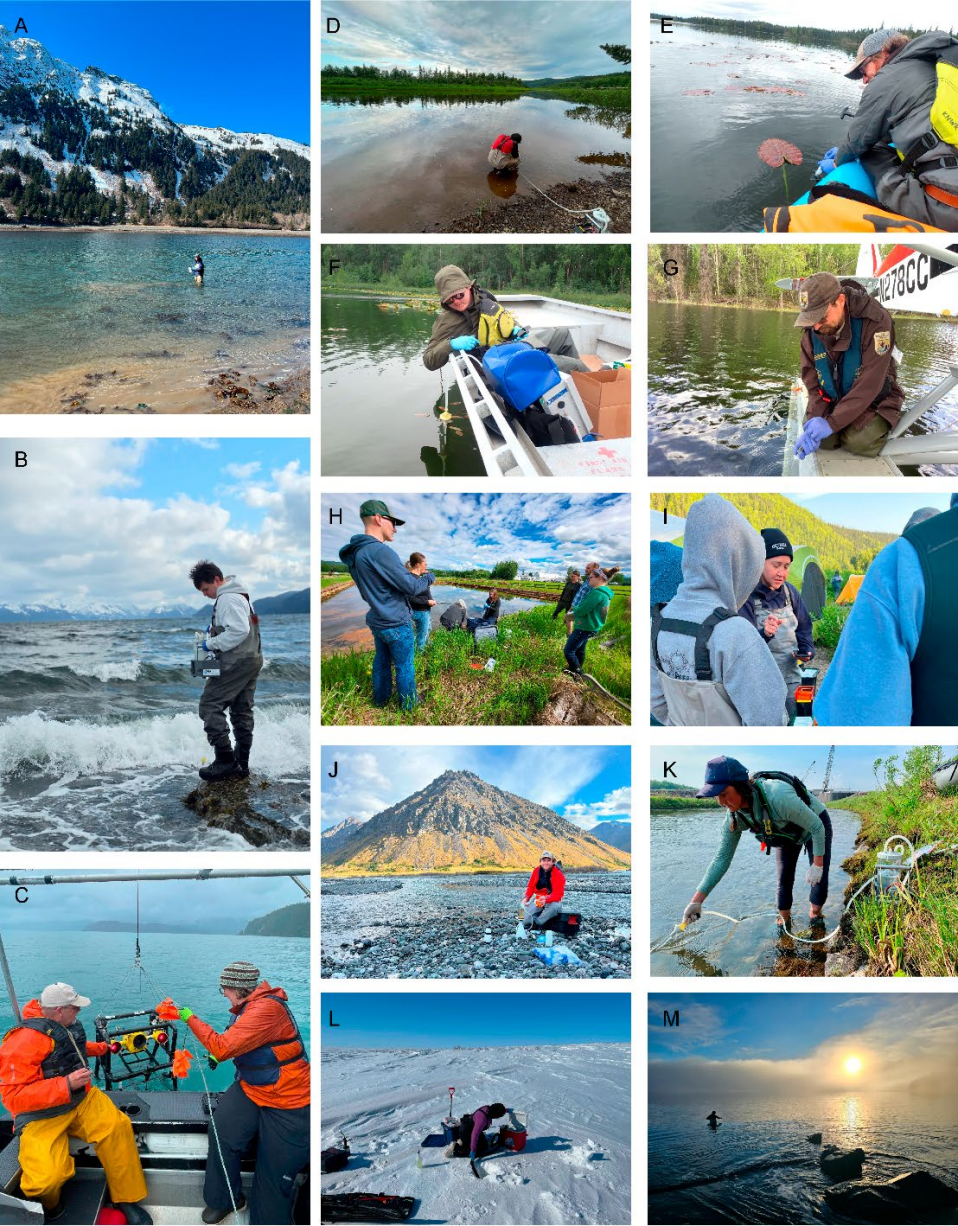
Jessica Glass in Kachemak Bay (credit: Lindsey Stadler/UAF); (B) Dustin Carl at Lowell Point in Resurrection Bay collects eDNA samples to detect fishes (credit: Allison Carl/Alutiiq Prime Marine Institute); (C) Markus Horning (Wildlife Technology Frontiers) and Amy Bishop with PESCA sampler (UAA) (credit: Jessica Glass/UAF); (D) Richie Wachter collects an eDNA sample at Takotna River to detect fishes (credit: Andrew Magel/Kuskokwim River Intertribal Fish Commission); (E) Steve Hoekwater collects an eDNA sample at Berg Lake to survey for fishes (credit: Nathan Davis/USFWS); (F) Nathan Davis collects an eDNA sample at Dolly Varden Lake to survey for fishes (credit: Matt Bowser/USFWS); (G) Matt Bowser filters an eDNA water sample from Barabara Lake to survey for fishes (credit: Dom Watts/USFWS); (H) UAF trains ADF&G personnel in eDNA sampling techniques to prepare for a field season in Northwestern Alaska (credit: Maggie Harings/UAF); (I) Brandi Kamermans (SALMONg LLC/UAF) teaches a field‐based eDNA course on the Kuskokwim River (credit: Erik Schoen/UAF); (J) Sebastian Zavoico at Whale Mountain on the Kongakut River collects eDNA to detect mammals, aquatic invertebrate communities, and fishes (credit: Ken Tape/UAF); (K) Maggie Harings collects an eDNA sample from the Chena River to detect and fishes (credit: Erik Schoen/UAF); (L) Katie Drew (BLM) collects samples at Harry Potter Lake; (M) Allison Carl at Lowell Point in Resurrection Bay (credit: Dustin Carl/Alutiiq Pride Marine Institute).

**FIGURE 4:**
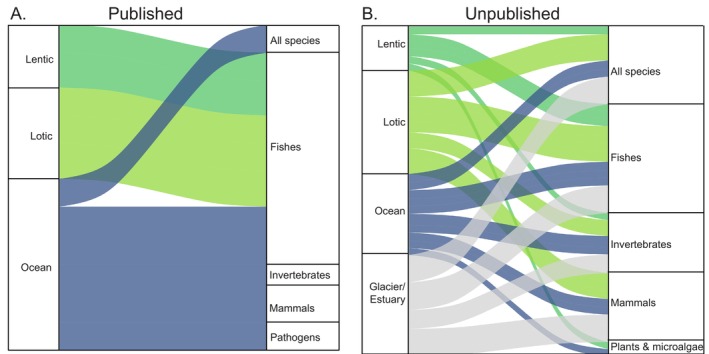
Alluvial plots comparing ecosystems to target taxa in (A) published and (B) unpublished studies.

Spatial data from published and unpublished studies reveal that projects mainly occurred along accessible locations (e.g., road systems, shorelines, and locations associated with scientific research surveys, such as the Northern Gulf of Alaska Long‐Term Ecological Research program) (Figure 2). These studies included a diverse array of environments, taxa, and sampling devices (Figure 3). While published literature was dominated by studies of fishes in lentic and lotic systems, unpublished research targeted a broader diversity of taxa, with some inclusion of mammals, invertebrates, and pathogens (Figure 4). Of all the aquatic ecosystems sampled, marine studies targeted the greatest taxonomic diversity (Deeg et al. 2023; Larson et al. 2022; Menning, Ward, et al. 2020; Menning et al. 2021; NOAA PMEL et al. 2023; Parsons et al. 2018). The fewest studies were conducted in the Bering Sea/Chukchi Sea (Bering, Chukchi, and Beaufort Sea; 1 response) and the Gulf of Alaska (1 response; Figure 9B).

**FIGURE 5:**
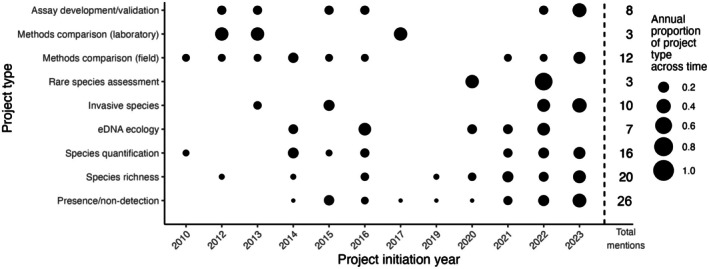
The bubble plot displays the number of combined published and unpublished eDNA studies as the proportion of eDNA project types applied between 2010 and 2023. For each category, the bubbles represent the proportion of projects started in each year.

The review and survey also revealed substantial variation in fundamental methodological research and in the methods applied. Methodological studies included those focused on assay development and validation (*n* = 8), field methods (*n* = 12), and laboratory methods (*n* = 3) (Figure 5). Most studies assessed presence/non‐detection of species (*n* = 26), species richness (*n* = 20), or species quantification (*n* = 16).

**FIGURE 6:**
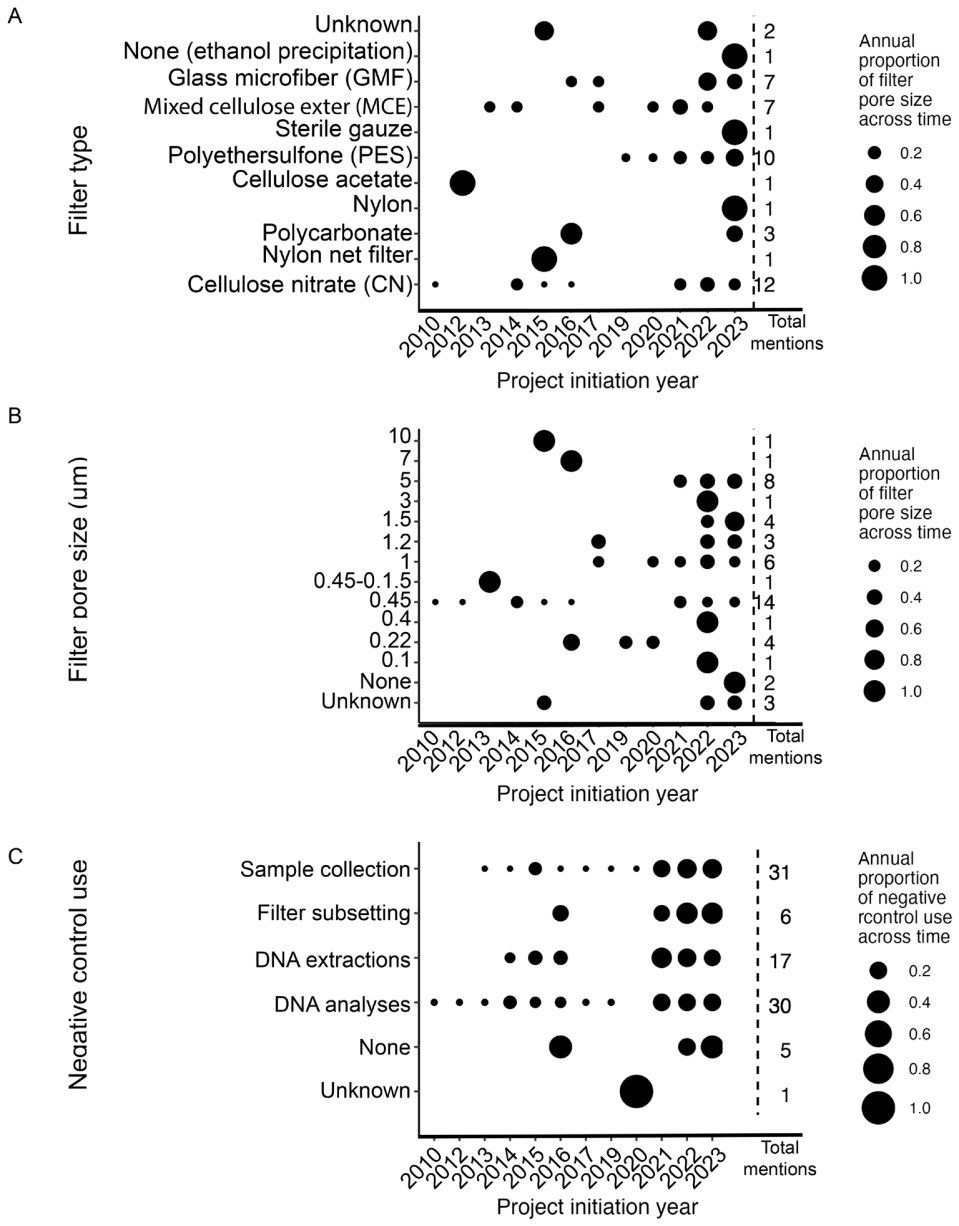
We assessed temporal trends in filter pore size, filter type, as well as the implementation of negative controls throughout the duration of the study (e.g., field negative, extraction negative). Bubble plots for (A) study types; (B) filter types; and (C) the use of negative controls at various steps during the sampling period between 2010 and 2023.

**FIGURE 7:**
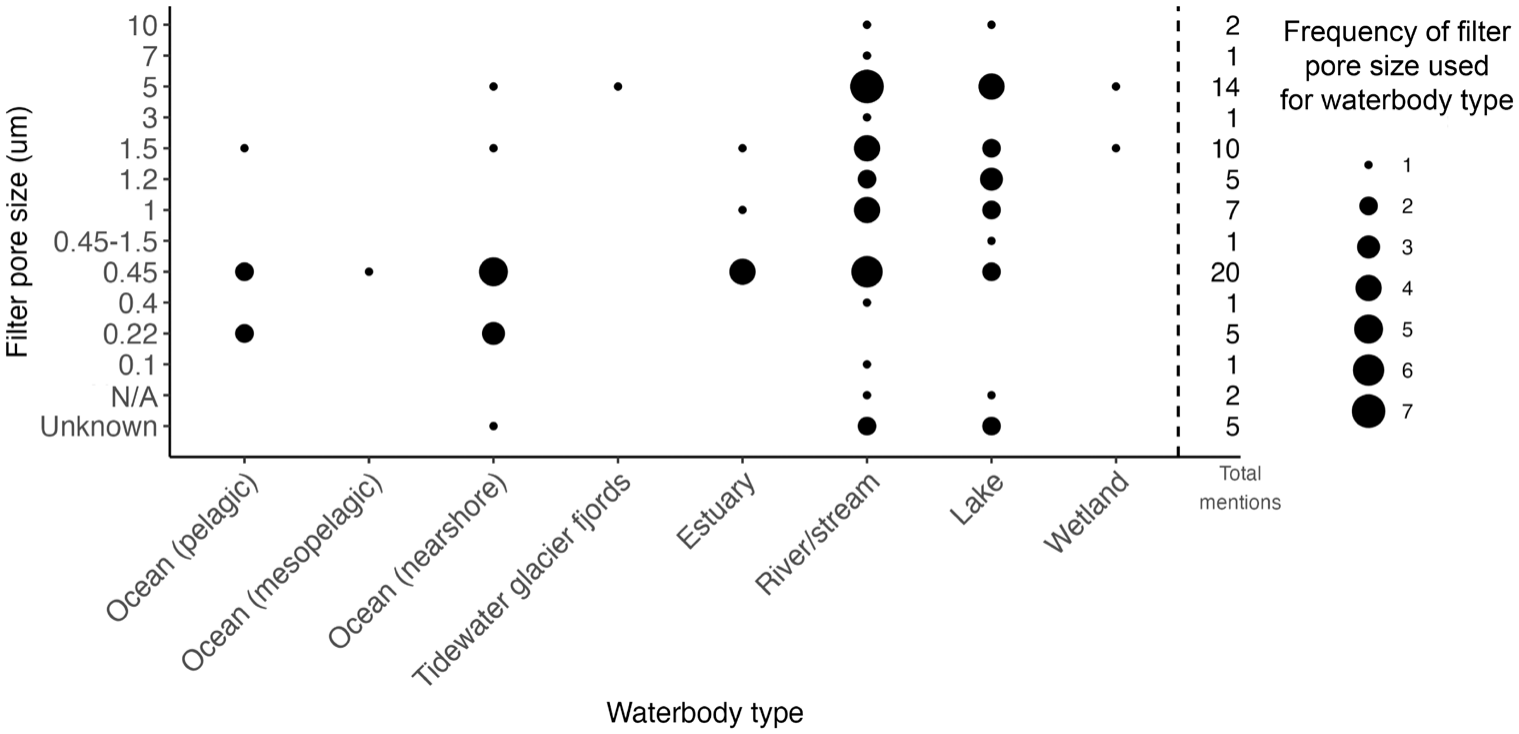
Bubble plots showing the distribution of filter pore sizes used across different waterbody types. Bubble size represents the frequency with which a given filter pore size was reported for each waterbody type. The “Total mentions” column indicates the number of times each filter pore size was reported across all studies for the ecosystem type.

**FIGURE 8:**
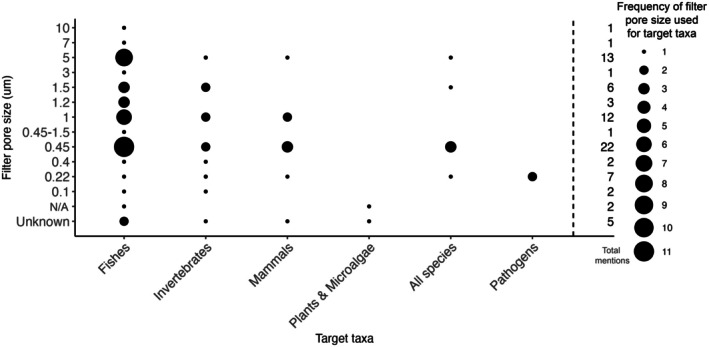
Bubble plots showing the distribution of filter pore sizes used across different target taxa. Bubble size represents the frequency with which a given filter pore size was reported for each target taxon. The “Total mentions” column indicates the number of times each filter pore size was reported across all studies for target taxa.

During 2010–2025, eDNA studies in Alaska used 14 unique filter pore sizes and 11 unique filter types (Figures 6, 7, 8 and Table A4). The three most‐commonly used filter pore sizes were 0.45, 1, and 5 μm, though the use of 5 μm filters did not begin until 2021. The diversity in filter pore sizes used across studies increased from 1 in 2010 to 9 in 2022 (Figure 6). Cellulose nitrate (*n* = 12), polyethersulfone (*n* = 10), and glass microfiber (*n* = 7) were the most frequently used filter types (Figure 6A).

We identified four stages throughout the process of eDNA application when practitioners used negative controls: (1) sample collection, (2) filter subsetting, (3) DNA extraction, and (4) DNA analyses (plate and library preparation; Figure 6C). Initially, negative controls were used solely during eDNA sequencing. Negative control use has increased over time, and only a small number of studies reported no negative controls or unknown implementation of negative controls. During more recent years (2021–2023), negative controls were implemented evenly across all four stages. Negative control implementation at sample collection was first reported in 2013 and has since increased in use, while filter subsetting and DNA extraction blanks were first reported in 2014 and 2016, respectively. Both practices were unreported between 2017 and 2020, only reappearing in studies in 2021.

**FIGURE 9:**
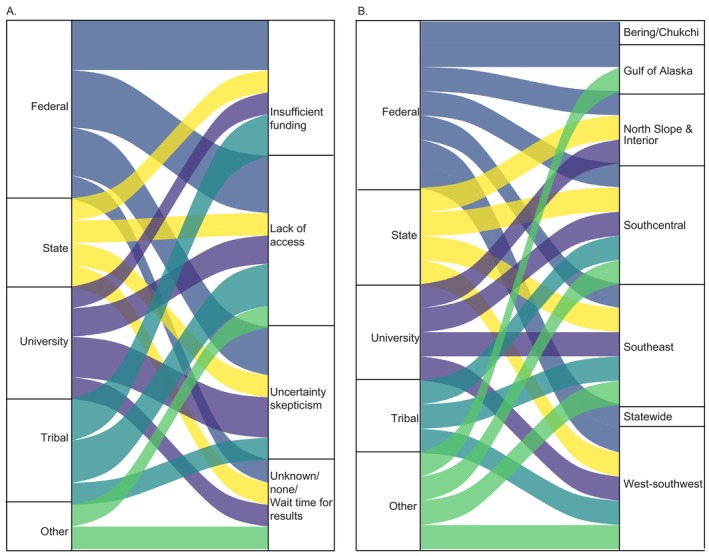
Alluvial plots illustrating the funding sources associated with (A) barriers faced while conducting eDNA research; (B) the Alaskan study regions from Figure 3. Other includes funding agencies that are international, private, pursuing funding, and unknown.

The eDNA‐based research and monitoring studies in Alaska were funded by federal and state agencies, private entities, tribal entities, universities, international organizations, and unknown funding sources. Federal funding (40 responses) was the most frequently reported, followed by state (29 responses) and university funding (11 responses). Southeast and Southcentral Alaska have the broadest sources of funding support. Tribally funded projects (4 responses) were limited in geographic scope, focusing on regions that included Southcentral, Southeast, and West‐Southwest. State‐ and university‐funded projects included the North slope and interior, Southcentral, Southeast, and West‐southwest regions (Figure 9B).

Survey participants identified unique barriers they faced while conducting eDNA research in Alaska (Figure 9). Within the survey the following options were provided: (1) insufficient funding, (2) lack of agency/organization support, (3) lack of laboratory access/lack of funding for sample analyses, (4) uncertainty about how to analyze data, (5) None—I am well‐versed in eDNA methods and applications, and (6) “other.” Barriers provided by respondents in “other” included the following: uncertainty about lab selection, skepticism from end‐users, inconclusive results, remote sampling locations, facility infrastructure, sampling equipment, laboratory equipment, existing assays specific to Alaskan species or regions, personnel trained in field and laboratory techniques, and organizational laboratory capacity. We binned these barriers into four categories. Binning was based on commonalities among barriers we included in the survey and barriers provided in “other” by survey participants. Insufficient funding, lack of access, uncertainty and skepticism about protocols, and unknown, none, and wait time for results were the top barrier groupings faced in descending order of mentions (Figure 9A).

**FIGURE 10:**
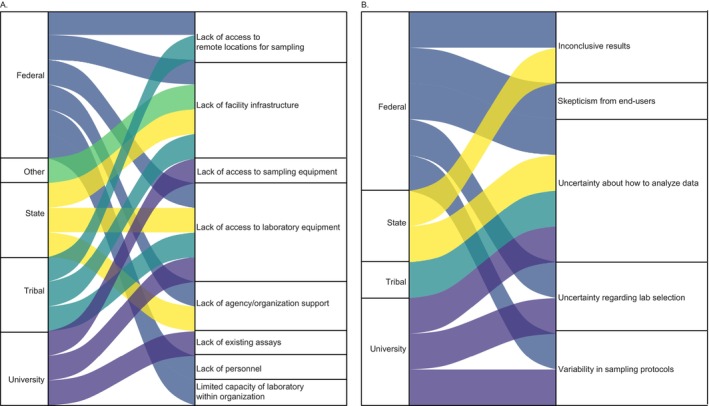
Alluvial plots illustrating the funding sources associated with two categories of barriers identified in Figure 6: (A) lack of access and (B) uncertainty and skepticism.

Lack of access included responses that spanned multiple funding sources. To provide insight into the lack of access bins, we provide an additional graph for the following barriers that we placed within this category: access to remote locations for sampling, lack of facility infrastructure, lack of access to laboratory equipment, lack of agency/organization support, lack of personnel, and limited capacity of laboratory within organization (Figure 10A). Federally funded projects identified lack of access to remote locations for sampling, lack of facility infrastructure, lack of access to laboratory equipment, lack of agency/organization support, lack of personnel, and limited capacity of laboratory within organization. State funded projects identified lack of facility infrastructure, lack of access to laboratory equipment, and lack of agency/organization support. University‐funded projects identified lack of access to sampling equipment, lack of access to laboratory equipment, and lack of existing assays. Tribally funded researchers identified lack of access to remote locations, lack of facility infrastructure, and lack of laboratory equipment. Funding agencies that included international, private, pursuing funding, and unknown were in the “other” category and identified only lack of facility infrastructure.

“Uncertainty and skepticism” barriers were binned together and we provided an additional graph that displays results for the barriers that were binned in this category: inconclusive results, skepticism from end‐users, uncertainty about how to analyze data, uncertainty about laboratory selection, variability in sampling protocols (Figure 10B). Federally funded projects had the most barriers in the “uncertainty and skepticism” category. The federally funded projects identified inconclusive results, skepticism from end‐users, uncertainty about how to analyze data, uncertainty regarding lab selection, and variability in sampling protocols. University‐funded researchers mentioned uncertainty about how to analyze data, uncertainty regarding laboratory selection, and variability in sampling protocols. State funded researchers identified inconclusive results and uncertainty about how to analyze data. Tribally funded researchers identified only uncertainty about how to analyze data.

**FIGURE 11:**
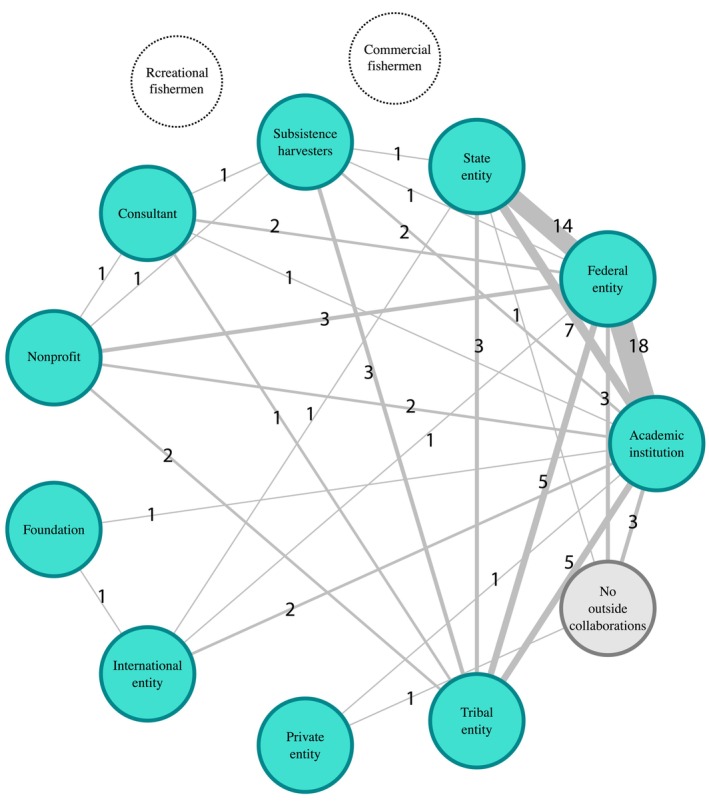
Network diagram for collaborations among eDNA researchers identified from the literature review and survey responses. Gray lines indicate a collaboration between entities, with line thickness representing the number of occurrences. Collaboration network among entities involved in eDNA research in Alaska, based on the literature review and survey responses.

Survey participants identified a broad range of collaborations in project development and implementation. Federal agencies (33 responses), academic institutions (27 responses), and state agencies (27 responses) were the most mentioned collaborators. One survey participant chose “other” and added “foundation.” Federal, state, and academic institutions were revealed as central hubs of collaboration, with state agencies and tribal entities also strongly connected (Figure 11). Other groups (e.g., nonprofits, private entities, subsistence harvesters) appeared less frequently but still formed part of the broader network. Inclusion of subsistence harvesters, and commercial and recreational fishermen revealed additional partnerships with local communities.

## 4 Discussion

This review highlights past and current applications of eDNA for aquatic biomonitoring in Alaska, revealing important advances in the field as well as key obstacles to fully implementing this technology in high‐latitude, remote regions. Our review represents 53 studies, published and unpublished, spanning aquatic ecosystems across Alaska and associated state and federal marine waters, dating back to 2010.

### 4.1 Application of eDNA in Alaskan Aquatic Biomonitoring

The increase in eDNA studies across the state over time is likely fueled by advancements in next‐generation sequencing and eDNA collection technologies, lower sequencing and data processing costs, and computational advancements in analyzing large ‐omics datasets. Comparable growth patterns have been documented in global eDNA reviews (Sahu et al. 2025; Takahashi et al. 2023). Notably, the application of metabarcoding is increasing in Alaska, mirrored globally by an increase in metabarcoding as an emerging and most widely used eDNA technique (Nørgaard et al. 2021; Sahu et al. 2025; Takahashi et al. 2023).

Globally, fishes and freshwater habitats are among the most frequently studied using metabarcoding (Capurso et al. 2023). Studies in Alaska mirror this, with a dominant research focus on fishes. The aquatic ecosystem sampled with the greatest taxonomic diversity targeted was the ocean (Deeg et al. 2023; Larson et al. 2022; Menning, Ward, et al. 2020; Menning et al. 2021; NOAA PMEL et al. 2023; Parsons et al. 2018). However, unpublished results revealed that the taxonomic breadth of eDNA research in Alaska extends well beyond that which is represented in the published literature (Figure 4).

### 4.2 Using eDNA for Fisheries Management

The emphasis of Alaskan eDNA studies on fishes likely reflects the cultural, ecological, and economic significance of Alaskan fisheries. The first wholesale value of commercial fisheries in Alaska's state waters and surrounding federal waters has fluctuated between roughly 2.5 billion – 6 billion US dollars between 2003–2023 (NOAA 2024). Alaska's vast and remote geography makes representative sampling for fisheries logistically difficult. These data gaps carry serious implications and population declines, and some Western Alaskan salmon stocks have resulted in fishery closures, heightened user conflicts, and profound cultural and food security impacts for Indigenous communities (Schoen et al. 2023). Researchers demonstrated that eDNA methods can complement out‐migrating smolt and returning adults at fisheries monitoring stations like the Auke Creek research weir in Juneau, Alaska (Levi et al. 2019). eDNA can also be used to track Chinook Salmon ( *Oncorhynchus tshawytscha* ) in natural environments (e.g., the Chena River, Alaska) (Matter et al. 2018) and sockeye salmon ( *Oncorhynchus nerka* ) in small streams (e.g., Hansen Creek, Alaska; Tillotson et al. 2018). In these studies, eDNA was proven to be promising for determining presence but not abundance of salmon. To quantify relative abundance of individuals, eDNA methods necessitate daily sampling and robust qPCR assay performance (e.g., 100% efficiency) (Matter et al. 2018). One of the strongest examples of eDNA applied to long‐term fisheries monitoring comes from the Chilkoot Indian Association, which has pioneered the use of eDNA for monitoring the spawning abundance of eulachon ( *Thaleichthys pacificus* ; Tlingit: [Saak]) since 2014 (Pochardt et al. 2020). The success of this program has allowed the tribe to discontinue a more costly and invasive mark‐recapture monitoring program and expanded efforts to monitor other fish populations and species using eDNA.

### 4.3 Data Gaps and Biomonitoring of Taxa Beyond Fishes

Notably, our dataset indicates a paucity of eDNA studies targeting aquatic bacteria and fungi in both published and unpublished literature in Alaska, which may have been due to the fisheries‐oriented audiences targeted by our survey. Our survey results from unpublished studies indicate an increased emphasis on invertebrates such as crustaceans and mollusks. Prior reviews of eDNA research suggest that microbial communities, microalgae, crustaceans, and mollusks are consistently underrepresented in published literature compared to taxonomic groups like fishes and mammals (Sahu et al. 2025). Applying eDNA methods to these groups presents specific challenges, including the difficulty of detecting intracellular microbes and the need for customized sampling strategies in highly turbid or structurally complex environments (Turba et al. 2023).

### 4.4 Insufficient Funding and Constraints on eDNA Research Design

Published literature and survey responses indicate that research efforts in Alaska have begun to address a broader diversity of study designs, ecosystems, and target species, laying important groundwork for developing regionally appropriate methodological standards. Despite this progress, researchers funded by federal, state, academic, and tribal entities consistently identified three major barriers to advancing eDNA work in Alaska: (1) insufficient funding, (2) limited access to resources, and (3) uncertainty or skepticism around protocols. These challenges are particularly pressing in eDNA, a field increasingly shaped by diverse interests and priorities across researchers, managers, and communities. The most recurrent perceived limitation identified by survey participants that impedes research design is funding constraints. Results are similar to those from eDNA survey results in 2023 (Capurso et al. 2023). Costs can be significant for the initial investment in eDNA research due to specialized equipment, reagents, and the need for trained personnel for sample collection, as well as laboratory and data analysis (Stein et al. 2024). Our survey reveals a lack of laboratory capacity and infrastructure for eDNA processing in Alaska. Respondents indicated there was limited access to remote locations, facility infrastructure, sampling equipment, agency and organizational support, species‐specific assays, personnel, and laboratories with processing capacity (Figure 10A). Without funding to provide training and equipped laboratories, as well as sustained institutional support, it will remain difficult for researchers employing eDNA approaches to generate enough data to alleviate ongoing uncertainty and skepticism about eDNA methods in Alaska (see Section 4.6).

Another major barrier is the absence of sequencing infrastructure within the state. Due in part to minimal in‐state sequencing facilities, many eDNA projects rely on private laboratories outside of Alaska to perform these essential laboratory methods (Table A5). For example, only seven institutions in Alaska have qPCR capabilities. These include the University of Alaska Fairbanks (UAF) and Anchorage (UAA) campuses (academic); United States Geological Survey (USGS), National Oceanographic and Atmospheric Administration (NOAA), and United States Fish and Wildlife Service (USFWS) (federal); ADFG (Alaska Department of Fish & Game) (state); and the Alutiiq Pride Marine Institute (tribal). Metabarcoding, which requires high‐throughput short‐read sequencing, can only be accomplished at five in‐state institutions and, of these, only the UAF Genomics Core Lab operates as a contract facility capable of conducting DNA extractions, amplification, and sequencing on an Illumina MiSeq. While federal collaborators generally have access to the necessary equipment and facilities, the other nine collaborators in the network reported limited or no access (Figure 10). In addition, we compiled a noncomprehensive list of companies offering eDNA analyses throughout the world (Table A5).

Hirsch et al. (2024) note that shipping can be a major constraint on access and research capacity in Africa, South America, and the Pacific Islands; a similar constraint is observed in Alaska. Laboratory supplies can take weeks to arrive in some locations due to lack of regional suppliers and statewide transportation infrastructure. This barrier makes it difficult for researchers to meet stringent standards or produce results in a timely manner. While sending samples out for processing may be less costly than acquiring materials and establishing local processing capacity, this approach raises additional challenges, including reduced control over experimental procedures and data handling. Moreover, only a subset of companies advertises data interpretation services, which remains one of the primary barriers for eDNA researchers in Alaska. Even when interpretation support is available, the cost of these services may be prohibitive for user groups already constrained by limited funding. This highlights a critical need for additional private or contract laboratories within Alaska that can support eDNA sample processing, sequencing, analysis, and methods development.

### 4.5 Region‐Specific Assays and Invasive Species Management

Our study highlights a lack of local species DNA reference libraries, which has been previously documented in several locations across Africa (Perry et al. 2022; Von Der Heyden 2023) and worldwide (Schenekar 2023). Relying on assays not specific to Alaskan species or populations introduces the risk of false positives or erroneously identifying a species. It can also lead to false negatives that fail to detect a present species. Species‐specific assays are especially valuable for the early detection of climate‐driven ecological changes, including harmful algal blooms and invasive species. For example, *Alexandrium* and *Pseudo‐nitzschia* algae show considerable genetic variation across environments and regions, spanning national and international boundaries (Brunson et al. 2024). Similarly, monitoring invasive species requires sensitivity to genetic variation within populations. Capturing resolution through eDNA assays would not only support detection but also provide insight into population structure, invasion pathways, and adaptive differences across environments (Coyle et al. 2019). Improving the sensitivity and accuracy of eDNA detection strengthens the reliability of ecological monitoring and management outcomes (Bohmann et al. 2022; Johnson et al. 2024; Rishan et al. 2024). To‐date, the Borealis Biodesign LLC (Menning 2025) is the only publicly available reference library that is annotated for Alaska‐specific assays, with a focus on fishes. Included in this review is a noncomprehensive list of primers and loci that have been used for eDNA analyses in Alaska (Table A6).

### 4.6 Uncertainty and Skepticism as a Perceived Barrier

Participants added additional barriers that included inconclusive results, skepticism from end‐users, and uncertainty about lab selection. No survey participant chose the option “I am well versed in eDNA methods and applications.” When asked about barriers, researchers identified uncertainty related to how to analyze samples and select suitable laboratories for data processing or analysis. Underlying both inconclusive results and skepticism from end‐users is uncertainty about using eDNA as a realistic, implementable tool for management. These barriers are faced by researchers on an international level and they impede the transition from research‐driven science to management (Stein et al. 2024; Thivierge et al. 2026), despite progress made in proof of concept studies within Alaska (Tillotson et al. 2018; Matter et al. 2018; Levi et al. 2019; Pochardt et al. 2020) (see also Section 4.2). Perceived limitations of eDNA such as uncertainty regarding data analysis and lab selection are related to a lack of standardized communication and training practices among scientists, managers, and policymakers. Additionally, limited access to computational resources and the specialized training required for bioinformatics present practical barriers, making eDNA technologies difficult to implement for many researchers (Hirsch et al. 2024). Researchers in Alaska have implemented coproduction research and knowledge exchange networks to mitigate barriers faced by partners (see Section 4.8). Moreover, the development and sharing of citable sampling protocols (Harings et al. 2024) has been initiated by some authors of this article to help demystify and standardize sampling methods across organizations and research teams.

### 4.7 Surmounting Barriers Through Standardization

As we embark on the potential use of eDNA across ecosystems and research collaborations, an emerging need to standardize field and laboratory methods has become evident (Çevik and Çevik 2025). Seasonal variability in biological and physical factors also influences eDNA detectability and persistence, underscoring the need for standardized sampling strategies to improve comparability across space and time (Morrison et al. 2026). Although a statewide genetics policy exists to protect wild populations in Alaska, primarily salmon (Davis and Burkett 1989), no formal accreditation currently exists for eDNA analyses under recognized organizations such as the International Organization for Standardization (Trujillo‐González et al. 2021). Furthermore, there is a lack of national oversight through agency‐specific guidelines. Solutions include new standardization and best practices guidelines (Cunningham et al. 2024; DeHart et al. 2023; Doi et al. 2021; Jeunen et al. 2019; Theroux et al. 2025). Despite limited guidance and widespread uncertainty, our study suggests Alaska‐based eDNA research conducted in recent years aligns well with current eDNA standards and guidelines, particularly in the consistent use of negative controls (Figure 6C). Alaska‐based eDNA studies demonstrate thoughtful implementation of quality assurance measures and are contributing valuable data to large, public databases. The studies included in our review can serve as guidelines for the application of eDNA in remote and extreme understudied habitats, where researchers face significant methodological hurdles and logistical constraints (Harper et al. 2019; Hirsch et al. 2024; Sanches and Schreier 2020).

We are unable to investigate how much variability exists in the amount of funding that is distributed among regions and specific study types. However, we can determine the diversity in funding types distributed across the state and surrounding marine ecosystems. For example, tribal agencies supported the fewest regions. Whereas, state, federal, and university‐funded studies were spread across all regions. The Bering, Chukchi, and Beaufort Seas were the fewest in number and funded by the fewest diversity of agencies. Future studies could examine if patterns like these and unequal agency support contribute to specific barriers for regions. For example, a series of survey questions could explore bias toward well‐funded research centers. Or a study could investigate if certain studies receive great support because they are focused on fundamental research questions. For instance, protocol development and “ecology” of eDNA are underrepresented in the global South or tropics (Schenekar 2023). Here, we would need more information to determine if funds are evenly distributed among institutions and studies.

Insufficient funding has been associated with a lack of access to standardization (Hirsch et al. 2024). We summarized guidelines (or lack thereof) in publications from the United States Department of Agriculture, USFWS, and USGS (Table A7). One of the first steps in designing an eDNA study is determining how samples will be concentrated, including filter type and pore size. Some survey participants reported uncertainty about variable sampling protocols and, indeed, we observed wide ranges of pore sizes and filter types used across studies. We also observed a wide range of filter pore sizes and filter types in publicly available guidelines, allowing flexibility in filter type and choice. We found that more studies that assess compatibility between filter types and pore size are warranted in Alaska. Our literature review and survey identified that cellulose nitrate or polyethersulfone filter types were the most common and that filter pore size selection among eDNA practitioners varied across time, ecosystems, and target taxa. Filter pore size can affect eDNA collection in different aquatic environments and waterbody types (Liu et al. 2024). The majority of eDNA in Alaska is being collected on 0.45 μm filters, regardless of differences in the targeted taxa or ecosystem type. It was beyond our study design to infer the reasoning behind the various filter types used. Future research could investigate the process and reasoning for filter selection for the various environments and ecosystems tested across the state.

### 4.8 Community‐Driven Needs, Promoting Coproduction

Coproduction of knowledge is a collaborative process that brings together diverse perspectives from researchers, agencies, and community partners to achieve shared research goals or products (Rudolf et al. 2025). In our evaluation of agencies and networks, we uncovered multiple collaborations with subsistence harvesters (Figure 11). Our survey also unveiled community‐driven monitoring efforts involving the Tanana Chiefs Conference, ADF&G, Kuskokwim River Intertribal Fish Commission, USFWS, Chilkoot Indian Association, and the Alutiiq Pride Marine Institute.

Knowledge sharing about eDNA collection and analytical methods has already begun between organizations and agencies in Alaska. eDNA studies on the Yukon and Kuskokwim rivers, initiated by tribal organizations and involving agency and academic partners, are underway (Figure 3D). Similarly, the Alutiiq Pride Marine Institute and researchers at the University of Alaska Fairbanks have developed a project‐specific research and data management plan that incorporates both the FAIR and CARE principles (Carroll et al. 2021). Training opportunities that are written into funding opportunities increase the sustainability of long‐term monitoring and ensure continuity in data collection. Sovereign Autonomy for Monitoring Non‐human genes (SALMONg LLC) conducts molecular‐based genetics education for environmental monitoring with tribal communities in Alaska (Figure 3I). Coproduction approaches, rooted in local priorities for natural resources, can provide equitable pathways to expand and sustain community‐led eDNA research.

While we did not explicitly ask about community‐driven monitoring needs in Alaska, it was evident that such partnerships inherently led to novel solutions to some of the barriers we uncovered, including working in highly remote settings, laboratory access, data management, and training. Future studies would benefit from surveying the specific entities involved in each stage of eDNA projects, the rationale behind methodological choices, challenges encountered, and project management strategies for successful implementation. Documenting these elements would enhance transparency, highlight how researchers and community members collaborate to overcome challenges, and provide a valuable resource for others seeking to develop community‐engaged monitoring programs. The effective use of eDNA in Arctic environments requires the capability to support community‐driven monitoring, equitable access, and locally led research initiatives. A previous study explored the capabilities, opportunities, and barriers of community‐based environmental monitoring programs, although eDNA applications were not specifically addressed (Danielsen 2020). Future work on this topic could target the effectiveness of community‐driven eDNA study designs with an emphasis on sample collection and frequency. For example, research about water quality studies compared the effectiveness of samples collected by community members to professional scientists in the Yukon River Basin, Alaska (Herman‐Mercer et al. 2018).

Stronger collaborative efforts could help build the foundation for applying eDNA to fisheries management and resilience planning in Alaska and other remote and data‐limited regions. eDNA is a revolutionary step in biodiversity monitoring; the growth and application of eDNA assessments fail to capture biodiversity in a geographically uniform fashion, with a marked prevalence of studies in the Global North (Hirsch et al. 2024; Takahashi et al. 2023). As on‐site genomic sequencing is becoming more feasible with portable, long‐read Oxford Nanopore Technology such as the MinION (Doorenspleet et al. 2025), partnerships with local researchers, research cruises, local communities, and agencies could provide opportunities to access water samples collected across more remote regions of Alaska, including open‐ocean environments.

In Alaska, aviation networks are the only means of year‐round transportation throughout the state, restricting research opportunities. Often, traveling to field sites requires researchers to have specialized skillsets such as operating ATVs, snowmachines, boats, or rafts (Figure 3E–G). In addition, skills required to fix equipment in the field are frequently needed, alongside those needed to collect and process samples. This necessitates strong relationships with local communities that have the expertise and equipment necessary to assist in sampling. These partnerships are not built on citizen science volunteer hours, but equitable funding opportunities that pay for salaries and resources for agencies to work collaboratively. For instance, workshops were designed and implemented by SALMONg LLC for the detection of species of interest in rural, western Alaska in the towns of Bethel and Aniak. In summer 2024, lessons were taught on a gravel bar upriver from the George River Weir, joined by members of the community and families of the students (Figure 3L). Battery‐powered generators were not allowed on the small aircraft that service Aniak and the team was able to access the Honda generator because the local communities had, over the course of 10 years, developed infrastructure with the Kuspuk School District and Math and Science Expedition. They built a program for remote teaching with the Native Village of Napaimute. Only by partnering with the Kuspuk School District, the Math and Science Expedition, and ADF&G did SALMONg have transportation and electricity to run the workshop.

In Alaska, geographic isolation leads to high shipping costs and limited in‐state infrastructure necessitates reliance on out‐of‐state laboratories, increasing costs and delaying data turnaround. Research is further hampered by logistical constraints in freezing temperatures, and researchers in Alaska have had to modify conventional eDNA sampling devices to prevent them from freezing during cold‐weather sampling events conducted via snowmachine, when many Arctic sites are more easily accessed via over‐snow travel. Specifically, researchers in freezing conditions have developed state‐of‐the‐art protocols to collect samples through ice (Figure 3L).

## 5 Key Limitations

The review we present is novel and informative, though we also recognize its limitations.

Key limitations of this review include its partial reliance on self‐reported survey data, potentially uneven taxonomic and regional coverage driven by the composition of survey participants, and the potential for publication bias within the available literature. The survey was distributed within our existing network of collaborators and contacts, which may have limited the breadth of perspectives represented. Additionally, responses may have been influenced by predefined multiple‐choice options, even though open‐ended “other” and negative “None” or “N/A” responses were also consistently provided. For example, target taxa categories were adapted from a global review (Takahashi et al. 2023) and may not fully capture the taxonomic representation of ongoing eDNA research in our study region. Despite these inherent limitations, the synthesis encompasses a large share of the relevant research conducted in Alaska, illuminating and helping advance a rapidly developing field of study in a remote, sparsely populated region that supports a tightly interconnected research community working in coordination to monitor and conserve highly valued aquatic resources.

## 6 Conclusions

Climate change is affecting biodiversity worldwide and in remote regions; eDNA monitoring has emerged as a promising tool to transform how we observe and measure biodiversity. We provide the first comprehensive spatial and temporal overview of aquatic eDNA monitoring in Alaska by reviewing publications and surveying eDNA scientists across the state. Our survey reveals that practitioners often struggle to identify appropriate sampling protocols and analysis methods due to the wide variety of approaches available and lack of existing standards. The application of eDNA at operational scales is constrained by a lack of laboratory facilities equipped to process eDNA samples, although several tribal organizations are actively developing their own laboratory spaces. Our review illustrates the extent to which researchers are facing barriers and forming collaborations, which can reduce some of these barriers.

In Alaska, government agencies, industry partners, academic institutions, and community organizations are advancing eDNA research and its application in understudied and changing ecosystems. Reliance on resources outside of the state (e.g., for DNA sequencing) raises important concerns regarding data sovereignty and governance, particularly given the large number of tribal communities in Alaska and the region's strong history of community‐based environmental monitoring, including over 170 programs across the Arctic. For communities and tribal organizations, limited local infrastructure restricts opportunities for locally driven research, workforce development, and the integration of eDNA tools into comanagement and subsistence monitoring frameworks. Addressing these gaps through investment in laboratory infrastructure, regional training programs, and locally accessible computational resources is essential for building sustainable, equitable eDNA capacity in Arctic and subarctic regions.

## Author Contributions

**Brandi Kamermans:** conceptualization (lead), data curation (lead), formal analysis (lead), funding acquisition (lead), investigation (lead), methodology (lead), project administration (lead), writing – original draft (lead). **Maggie Harings:** conceptualization (equal), data curation (equal), formal analysis (equal), funding acquisition (equal), investigation (supporting), methodology (supporting), project administration (supporting), writing – original draft (equal). **Rachel Lekanoff:** conceptualization (equal), data curation (supporting), formal analysis (supporting), investigation (supporting), methodology (supporting), writing – original draft (equal). **Laura E. Timm:** data curation (equal), formal analysis (equal), writing – original draft (equal). **Erik Schoen:** funding acquisition (equal), writing – review and editing (supporting). **J. Andrés López:** writing – review and editing (equal). **Jessica R. Glass:** funding acquisition (equal), methodology (supporting), writing – original draft (equal).

## Funding

This work was supported by the National Science Foundation (OIA‐2344553) and State of Alaska.

## Conflicts of Interest

The authors declare no conflicts of interest.

## Data Availability

De‐identified data supporting the findings of this study are provided in the Appendix. Data containing personally identifiable information are not available due to ethical restrictions and the need to protect participant anonymity, in accordance with informed consent agreements.

## Appendix

**TABLE A1:** Keywords used to identify and studies published using environmental DNA methods in Alaska.

Search engine	Keywords
Connected papers	Alaska, environmental, DNA
Google Scholar, PubMed, JStor, Research Gate, Semantic Scholar	Arctic, Alaska, environmental, DNA, quantitative PCR, qPCR, metagenomics, Taqman, digital droplet PCR, ddPCR, digital PCR, dPCR, metabarcoding, primers, high‐throughput, illumina, sequence, biodiversity, monitoring, invasive, endangered, salmon

**TABLE A2:** Publications from 2024 to 2025 that are not included in the data analyses and figures.

References	Project start year	Study type	Ecosystem	Target taxa	Region	Filter type	Filter pore size	DNA analyses
Baetscher et al. (2024)	2021	Quantification	Ocean	Chum salmon ( *Oncorhynchus keta* )	Southeast Alaska	Cellulose nitrate (CN)	0.45	qPCR
Benson et al. (2024)	2018	Presence/non‐detection, species quantification	Lake	Plants (waterweed *Elodea canadensis* and western waterweed *E. nuttallii* )	NA	Glass microfiber (GMF)	1.2	qPCR
Deeg et al. (2025)	2022	Presence/non‐detection	Ocean	*Oncorhynchus*	Northeast Pacific	0.45	PES	Metabarcoding
Gillespie et al. (2024)	2023	NA	Ocean	NA	Aleutian Islands, Gulf of Alaska, Unalaska	0.45	NA	NA
Ledger et al. (2025)	NA	Presence/non‐detection	Ocean	Fishes (*Sebastes* species)	AFSC Groundfish Assessment Program 2022 Aleutian Islands Bottom Trawl Survey	Cellulose nitrate (CN)	0.45	Metabarcoding
Ledger et al. (2024)	2022	Quantification	NA	Walleye pollock ( *Gadus chalcogrammus* ), Pacific cod ( *Gadus macrocephalus* ), and Arctic cod ( *Boreogadus saida* )	NA	NA	NA	qPCR and metabarcoding
Parsons et al. (2025)	2016	Presence/non‐detection	Ocean	Mammals (harbor porpoise [*Phocoena phoc‐oena*])	Inshore waters of Southeast Alaska and Western Gulf of Alaska	Mixed cellulose ester (MCE)	0.45	Metabarcoding

**TABLE A3:** Survey responses from 15 published articles. Responses were recorded for barriers, project region, funding, and project collaborators.

References	Barriers	Project region	Funding	Project collaborators
Matter et al. (2018)	None	Interior	Federal, State	State entity, Federal entity, Academic institution
Khalsa et al. (2020)	None	Interior	Federal, State	State entity, Federal entity, Academic institution
Levi et al. (2019)	Unknown	Southeast	Federal, State, International	State entity, Federal entity, Academic institution, International entity
Dunker et al. (2016)	Insufficient funding	Southcentral	Federal	State entity, Federal entity
Rodgers et al. (2018)	Unknown	North‐northwest	State, Federal, University	Federal entity, Academic institution
Menning, Simmons, and Talbot (2020)	Insufficient funding, Lack of personnel	Southcentral, Interior, North‐northwest	Federal	State entity, Federal entity, Academic institution
Menning et al. (2021)	Insufficient funding, lack of personnel	North‐northwest, West‐southwest	Federal	Federal entity
Menning, Ward, et al. (2020)	Insufficient funding, lack of personnel	West‐southwest	Federal	Federal entity
Deeg et al. (2023)	Unknown	Gulf of Alaska	International, Federal	Academic institution, Foundation, International entity
Pochardt et al. (2020)	Unknown	Southeast	State, Federal	Federal entity, Academic institution
Larson et al. (2022)	None	Southeast	Federal, State	State entity, Federal entity, Academic institution
Parsons et al. (2018)	None	Southeast	Federal	Federal entity, Nonprofit, Consultant
Galaska et al. (2023)	Access to remote locations for sampling	Bering Chukchi, Beaufort Sea	Federal	Federal entity
Sepulveda et al. (2018)	Skepticism from end‐users	Southcentral	Federal	State entity, Federal entity
Tillotson et al. (2018)	Uncertainty about how to analyze data	West‐southwest	Federal, University	Species quantification, eDNA ecology

**TABLE A4:** Survey responses from 15 published articles. Responses are recorded for study type, ecosystem, target taxa, filter type, filter pore size, and DNA analyses.

References	Study type	Ecosystem	Target taxa	Filter type	Filter pore size	DNA analyses
Matter et al. (2018)	Presence/non‐detection, species quantification, eDNA ecology, methods comparison (field)	River/stream	Fish (including lampreys)	Cellulose nitrate (CN)	0.45	qPCR
Khalsa et al. (2020)	Presence/non‐detection, species quantification, eDNA ecology, methods comparison (field)	River/stream	Fish (including lampreys)	Glass microfiber (GMF)	7	qPCR
Levi et al. (2019)	Species quantification, methods comparison (field)	River/stream	Fish (including lampreys)	Cellulose nitrate (CN)	0.45	qPCR
Dunker et al. (2016)	Invasive species, methods comparison (laboratory), assay development/validation, methods comparison (field)	Lake	Fish (including lampreys)	Mixed cellulose ester (MCE)	0.45–1.5	qPCR
Rodgers et al. (2018)	Assay development/validation, species quantification, invasive species, presence/non‐detection	River/stream, lake	Fish (including lampreys)	Nylon net filter	10	qPCR
Menning, Simmons, and Talbot (2020)	Species richness, assay development/validation, methods comparison (field), methods comparison (laboratory)	Lake, river/stream	Fish (including lampreys)	Cellulose acetate	0.45	Metabarcoding
Menning et al. (2021)	Species richness	Ocean (nearshore)	Pathogens	Polycarbonate (PC)	0.22	Metabarcoding
Menning, Ward, et al. (2020)	Species richness, assay development/validation, presence/non‐detection	Ocean (nearshore)	Pathogens	Polycarbonate (PC)	0.22	Metabarcoding
Deeg et al. (2023)	Species richness, presence/non‐detection	Ocean (pelagic)	Fish (including lampreys), macroinvertebrates, Cephalopods, Chordates	Polyethene sulfone (PES)	0.22	Metabarcoding
Pochardt et al. (2020)	Species quantification, methods comparison (field)	River/stream	Fish (including lampreys)	Cellulose nitrate (CN)	0.45	dPCR
Larson et al. (2022)	Presence/non‐detection, eDNA ecology	Ocean (nearshore)	Fish (including lampreys)	Cellulose nitrate (CN)	0.45	Metabarcoding
Parsons et al. (2018)	Presence/non‐detection, sequencing	Ocean (nearshore)	Mammals (marine)	Cellulose nitrate (CN)	0.45	Metabarcoding, qPCR
Galaska et al. (2023)	Species richness	Ocean (nearshore), Ocean (pelagic)	All species	Polyethene sulfone (PES)	0.22	Metabarcoding
Sepulveda et al. (2018)	Presence/non‐detection, methods comparison (laboratory)	Lake	Fish (including lampreys)	Glass microfiber (GMF), mixed cellulose ester (MCE)	1, 1.2	qPCR
Tillotson et al. (2018)	Species quantification, eDNA ecology	River/stream, lake	Fish (including lampreys)	Cellulose nitrate (CN)	0.45	qPCR

**TABLE A5:** A noncomprehensive list of companies offering eDNA analyses throughout the world, as well as the services they offer based on content appearing on their website. Note that many of these companies likely offer additional services beyond what was noted on their websites.

Company	Location	Field kits	Extractions	Library prep	Metabarcoding	q PCR	d PCR	dd PCR	Data analysis	Training
AllGenetics	Spain	×	×		×					
Applied DNA Sciences	United States									
Applied Genomics LTD	United Kingdom	×	×		×					
AquaBiota	Sweden				×	×				
Azenta/Genewiz	United States					×	×			
Borealis Biodesign	United States				×				×	
Bureau Veritas	North America					×				
Dante Genomics					×					
CALeDNA	United States	×	×	×	×	×		×		×
eDNA Labs	Croatia		×		×	×		×		
eDNAtech	Canada	×	×		×					
EnviroDNA	Australia				×	×				
Environmental Genomics and Conservation Genetics Laboratory	United States				×	×			×	
Eurofins Genomics					×	×				
Fera	United Kingdom	×								
Genidaqs	United States	×	×		×	×			×	
ID‐Gene	Europe	×	×		×				×	
Identifica	Portugal	×	×		×	?				
Jonah Ventures	United States	×	×		×	×				
Measurlabs	Finland				×					
National Genomics Center for Wildlife and Fish Conservation	United States									
Pacific Northwest Environmenal DNA Laboratory	United States		×			×			×	
SGS	Portugal		×		×					
SimplexDNA	USA, Europe	×	×		×					
Sinsoma	Austria	×	×		×					
SPYGEN	France, Canada	×			×					
SureScreen Scientifics	England	×				×				
Sylphium Molecular Ecology	Netherlands	×	×		×	×				
Taxus Medio Ambiente	Spain				×					
TropWATER	Australia		×		×				×	×
Wilder Lab	New Zealand	×			×					

*Note:* ? refers to the category being unknown based on the website search.

**TABLE A6:** Primer sets and probes for eDNA analysis used in Alaska.

Target taxa	5′–3′ end	Target region; taxa	Citation
Vertebrate specific; *Ophiodon elongatus* , *Stenobrachius leucotensis*, *Oxylebius pictus* , *Lipolagus ochotensis* , *Diaphus*, *Trichodon trichodon* , *Sebastinae*, *Atheresthes*, *Liparidea*, *Oligocottus maculosus* , *Gasterosteidae*, *Artedius*, *Anoplopomatidae*, *Hexagrammos*, *Osmeriformes*, *Ammodytes*, *Gadidea*, *Clinocottus Ccuticeps*, *Leptocottus armatus* , *Peuronectidae*, *Clupea pallasii* , *Cottiodidei*, *Salmoninae*	12S‐V5‐F CGACAGGTTCAGAGTTCTACAGTCCGACGATCACTGGGATTAGATACCCC 12S‐V5‐R GTGACTGGAGTTCAGACGTGTGCTCTTCCGATCTTAGAACAGGCTCCTCTAG	12S mitochondrial gene	Larson et al. (2022)
*Pleuronectidae*, *Onchorynchus nerka*, *Stenobrachius*, *Lipolagus ochetensis*, *Sebastes*, *Tarletonbeania*, *Squalus acanthias* , *Onchorynchus kisutsh*, *Oncorynchus keta*, *Symbolophorus californiensis* , *Onchorynchus gorbusha*, *Clupea pallasii* , *Lycodinae*, *Diaphus theta* , *Nansenia*		Cytochrome oxidase subunit 1 (COI)	Deeg et al. (2023)
*Esox lucius*	EluCOI‐F CCTTCCCCCGCATAAATAATATAA EluCOI‐R GTACCAGCACCAGCTTCAACAC EluCOI probe 6FAM‐CTTCTGACTTCTCCCC‐MBG‐NFQ	Cytochrome oxidase subunit 1 (COI)	Dunker et al. (2016)
*Esox lucius*	EluCOI‐F CCTTCCCCCGCATAAATAATATAA EluCOI‐R GTACCAGCACCAGCTTCAACAC EluCOI probe 6FAM‐CTTCTGACTTCTCCCC‐MBG‐NFQ	Cytochrome oxidase subunit 1 (COI)	Sepulveda et al. (2018)
*Oncorhynchus nerka*	F GGAAACCTTGCCCACGCG R AAAAGTGGGGTCTGGTACTGAG Probe FAM‐CTCTGTTGACTTAACCATC‐MGB	Cytochrome oxidase subunit 1 (COI)	Levi et al. (2019)
*Oncorhynchus kisutch*	F CGCTCTTCTAGGGGATGATC R CTCCGATCATAATCGGCATG Probe FAM‐ATTTACAACGTAATCGTC‐MGB	Cytochrome oxidase subunit 1 (COI)	Levi et al. (2019)
*Oncorhynchus tshawytscha*	F CTGGCACMGGGTGAACAGTCTACC R AAT GAA GGG AGA AGA TCG TYA GAT CA Probe 6FAM‐CTCCTGCGTGGGCTAG‐MBG‐NFQ	Cytochrome oxidase subunit 1 (COI)	Khalsa et al. (2020)
*Labyrinthula* sp.	F CAATGAATATCTTGGTTTCCG R GAGTGCTCGTTTGTGGACG	5.8	Menning et al. (2021)
*Labyrinthula* sp.	F ACCACATCCAAGGAAGGC R AATATACGCTACTGGAGC	18S	Menning et al. (2021)
*Halo/Phytophthora* spp.	F AACTTTCCACGTGAACCG R TAAAAGCAGAGACTTTCG	ITS	Menning et al. (2021)
*Phytophthora* sp.	F TCDTCDHTATTAGGTGC R GTRTTWAARTTTCTATC	Cytochrome oxidase subunit 1 (COI)	Menning, Ward, et al. (2020)
*Oncorhynchus* sp.	AK16SF CGA GAA GAC CCT ATG GAG C AK16SR GCG CTG TTA TCC CTA GGG T	16S	Menning, Simmons, and Talbot (2020)
*Oncorhynchus* sp.	AK12S‐F CTC GTG CCA GCC ACC GCG GTTA AK12S‐R GGG TAT CTA ATC CCR GTT TG	12S	Menning, Simmons, and Talbot (2020)
*Oncorhynchus* sp.	AKCOISal‐F TAG TAT TTG GTG CCT GAG C AKCOISal‐R ATY ATA ACG AAG GCA TGG GC	Cytochrome oxidase subunit 1 (COI)	Menning, Simmons, and Talbot (2020)
*Coregonus* sp.	AKCOICor‐F GCT GCT AGG ACA GGA AGG GA AKCOICor‐R GCT GCT AGG ACA GGA AGG GA	Cytochrome oxidase subunit 1 (COI)	Menning, Simmons, and Talbot (2020)
*Prosopium coulterii*	AKCOIProR ATC ATA ACG AAG GCG TGG GC	Cytochrome oxidase subunit 1 (COI)	Menning, Simmons, and Talbot (2020)
*Oncorhynchus nerka*	SECO3_861‐930‐F TCTGCCCTTCTCCTTACGATTTT SECO3_861‐930‐R GTTCGACCTAGAAATCGCCCTT SECO3_861‐930‐probe 6FAM‐5′‐CCATCCTGTTCCTCCT‐3′‐MGBNFQ	Cytochrome c oxidase subunit III gene	Tillotson et al. (2018)
Eukaryotes, bacteria and archaea	F GTGYCAGCMGCCGCGGTAA R CCGYCAATTYMTTTRAGTTT	16 rRNA mitochondrial locus	Galaska et al. (2023)
Eukaryotes and bacteria	F CTGGTGCCAGCAGCCGCGGYAA R TCCGTCAATTYCTTTAAGTT	18S nuclear RNA	Galaska et al. (2023)
*Thaleichthys pacificus*	5′‐Euc_COI_R‐F CTCCCTCCTTCCTTCTCCTT Euc_COI_R‐R GGTCTGGTACTGGGAAATGG Euc_COI_R‐Probe 6FAM‐AGCGGGAGCCGGGACTGGCT‐MGBNFQ	Cytochrome oxidase subunit 1 (COI)	Pochardt et al. (2020)
*Lota lota*	F GCCGTAATACTCCTTGGCCTT R CAATCGGGTTAGCGGGTGTA Probe FAM‐TGCCCTTGCCCTCTTCT‐	Cytochrome oxidase subunit 1 (COI)	Rodgers et al. (2018)
*Cottus cognatus*	F GGAGGCGTCCTAGCCCTC R GAGTCCAAAATAGGAATTGGGTCAC Probe FAM‐CATCCATCCTGGTGCTCAT‐MGB‐NFQ	Cytochrome b (cytb)	Rodgers et al. (2018)
*Salvelinus alpinus/Salvelinus malma*	F CCGCCACAGTACTTCACCTTCTA R AGGCCAAGCAATATAGCTACGAAA Probe FAM‐CCGACAAAATCTCATTCC ‐MGB‐NFQ	Cytochrome oxidase subunit 1 (COI)	Rodgers et al. (2018)
*Thymallus arcticus*	F TGTGGGCTGTTCTGATTACCG R TGCTGGGTCAAAGAAAGTGGTATTA Probe FAM‐CTTGCAGCAGGTATC‐ MGB‐NFQ	Cytochrome oxidase subunit 1 (COI)	Rodgers et al. (2018)
*Elodea* spp.	F GAAGCGGCAGAAATCAGTGG R TCTTGGGGTTTTMGTTATTTTGACCR Probe FAM‐TCATAGTAAACTAAGTTCCTCACAC‐MGB‐NFQ	Elod‐1	Benson et al. (2024)
*Elodea* spp.	F‐ TATCCAAAARGGTCCCGCCC R‐ TGCATCATGTTGAAACGCGA FAM‐TGCYTGCGTTACGTGAAAGTAATACGT ‐MGB‐NFQ	Elod‐2	Benson et al. (2024)
*Elodea canadensis*	F CCTATTGGCCAAGAATTCATTAAG R CTCTGATMGAATTGGAAAGCATTAAA Probe FAM‐CTTTTATGAAGTAGGGATATTCCATCG‐MGB‐NFQ	*ELCA7‐1*	Benson et al. (2024)
*Elodea nuttallii*	F CYTTGATTGGGCCCTCTCAG R GGTTGAAGATCACAGGGCGA Probe FAM‐ CCAAGAACCGAATTAAAAATAGAGTGG‐MGB‐NFQ	*ELNU2‐1*	Benson et al. (2024)
*Sebastes*	SebDLoop_F ATNACCATATCTAGGNTTNAACC SebDLoop_R TGRRCTTGTTGGTCGGYT		Ledger et al. (2025)
*Sebastes*	Thr‐RF GAGGAYAAAGCACTTGAATGAGC D‐RF CCTGAAAATAGGAACCAAATGCCAG		Ledger et al. (2025)

**TABLE A7:** Published guidelines used and published by resource management with summaries from each resource for what sampling methods are recommended. Methods include when and if to use negative controls, which filter pore size and filter type to use, and year of publication for eDNA‐based methods.

	Sampling method	Negative controls	Filter pore size	Filter type	Cite
United States Department of Agriculture	Vacuum filtration	Laboratory	1.5 μm	Glass microfiber	Carim et al. (2016)
United States Fish and Wildlife Service	Vacuum filtration, centrifugation, precipitation, flocculation	Field and laboratory	0.2–5 μm	Glass microfiber, cellulose nitrate, or mixed cellulose ester filters	Bockrath et al. (2022)
United States Geological Survey	Vacuum filtration	N/A	N/A	N/A	Laramie, Pilliod, Goldberg, and Strickler (2015)
Alaska Invasive Species Partnership	N/A	N/A	N/A	N/A	Dunker et al. (2022)
Alaska Department of Fish and Game Division of Sport Fish	Vacuum filtration	N/A	1.0–1.2 μm	Whatman glass	Massengill et al. (2022)
United States Geological Survey, Nonindigenous Aquatic Species Database	Vacuum filtration	Field and laboratory	N/A	N/A	Ferrante et al. (2023)

### Survey Questions

University of Alaska Fairbanks (UAF) staff and graduate students in the College of Fisheries and Ocean Sciences and the International Arctic Research Center are investigating how environmental DNA (eDNA) science is being applied to address research questions in the state. Specifically, they are interested in projects in *Alaska* that use *eDNA from water samples specifically (no soil samples, ancient DNA, stomach content samples, fecal samples, etc.)*. Many eDNA studies in the state are ongoing and have not yet been published, making a literature review limiting. In turn, UAF researchers developed this survey to gather additional data on unpublished projects. *If your project is published, you can use this survey to provide links to these documents (see “Survey guidelines”)*. The long‐term goal of this effort is to foster discussions of possible *standardization of eDNA methods and applications* to maximize the power of this ecological tool. Findings from this project will be presented at AMSS and AFS‐AK 2024 and will aid in the development of a review paper.

#### Survey Guidelines

- *Submit a single survey* if your projects have been published (peer‐reviewed or technical reports). When you begin the survey, you'll reach a question that allows you to share links or titles to all publications in a single entry, thus avoiding a submission for each study. *Time commitment: 5 min*.
- *Submit one survey per study* if your projects are unpublished (the survey goes faster than you may anticipate!). *Time commitment: 10 min*.

Feel free to forward this to other project leads that might be interested in contributing to this statewide project. *We look forward to sharing our findings with you!*

#### Statement of Consent

I understand the information presented to me. My questions have been answered to my satisfaction, and I agree to participate in this study. I am 18 years old or older and I have been offered a copy of the consent to participate form (click HERE to download). By clicking the “submit” button at the end of this survey I agree to participate.

#### Survey Questions

*Project status*

1. Project status
  - Completed, published (peer‐reviewed or technical report)
  - Completed, unpublished
  - Ongoing, unpublished
  - Other:
2. Publication link(s)
3. Please provide a link to your publication(s) and/or report(s) corresponding to your eDNA project(s)—we'll take care of the rest!
4. If you're willing, please provide a general description of your sampling location below (e.g., lake name, section of river, ocean zone, single lat/long in center of sampling locations, link to metadata with location(s), etc.). If you've submitted DNA sequence data to NCBI with metadata that contained sampling locations, you can include the corresponding links below.

*Project development*

5 When did you collect your first sample (MM‐YY, for example if you started in April 2023 you would answer 04‐23)?
6 When did you collect your last sample (MM‐YY)?
7 In what region of Alaska did your project take place? If marine‐based, please select “other” and enter the general location (e.g., Cook Inlet). If your study area lands on the border of two regions, select both regions.
  - 1 (north‐northwest)
  - 2 (interior)
  - 3 (west‐southwest)
  - 4 (southcentral)
  - 5 (southeast)
8 Which type(s) of collaborator(s) were involved with project development and/or implementation (select all that apply)?
  - State agency
  - Federal agency
  - Tribal entity
  - Academic institution
  - Nonprofit
  - Consultant
  - Subsistence harvesters
  - Recreational fishermen
  - Commercial fishermen
  - Other:
9 What was the primary funding source(s) for your study (select all that apply)?
  - Federal grant funding
  - State grant funding
  - Tribal grant funding
  - Private funding
  - Other:
10 What best describes your project (select all that apply)?
  - Biodiversity/species richness assessment
  - Invasive species assessment
  - Rare species assessment
  - Presence, non‐detection of specific species
  - Species quantification
  - eDNA ecology (distribution, persistence, etc.)
  - Field sampling methods comparison
  - Laboratory methods comparison
  - Assay development, validation
  - Other:

*Sample collection*

11 What time of water bodies did you collect your samples from (select all that apply)?
  - River/stream
  - Lake
  - Wetland
  - Estuary
  - Ocean (nearshore)
  - Ocean (pelagic)
  - Other:
12 What species were you targeting?
  - All species found in sample (biodiversity assessment)
  - Fish (including lampreys)
  - Crustaceans
  - Reptiles
  - Mammals (marine)
  - Mammals (land‐based)
  - Mollusks
  - Macroinvertebrates
  - Plants (macrophytes)
  - Microalgae
  - Other:
13 What technique(s) did you use to analyze your DNA extractions (select all that apply)?
  - Metabarcoding
  - qPCR (i.e., real‐time PCR)
  - Digital PCR (dPCR)
  - Droplet digital PCR (ddPCR)
  - CRISPR
  - Other:
14 What type of filter did you use during filtration (select all that apply)?
  - Mixed cellulose ester (MCE)
  - Polyethene sulfone (PES)
  - Polycarbonate (PC)
  - Cellulose nitrate (CN)
  - Polyvinylidene fluoride (PVDF)
  - Glass microfiber (GMF)
  - Other:
15 What was the filter pore size?

*Laboratory + data analyses*

16 At what steps did you implement biological and/or technical replicates (select all that apply)?
  - Field sampling
  - Filter subsetting (i.e., cutting filters in half—one half archived, the other used for DNA extractions)
  - DNA extractions
  - DNA analyses (metabarcoding, qPCR, etc.)
  - Other:
17 At what steps did you implement sample blanks (i.e., field blanks, qPCR blanks; select all that apply)?
  - Sample collection
  - Filter subsetting (i.e., cutting filters in half—one half archived, the other used for DNA extractions)
  - DNA extractions
  - DNA analyses (metabarcoding, qPCR, etc.)
  - Other:
18 Which of the following techniques did you use (select all that apply)?
  - Metabarcoding
  - qPCR (i.e., real‐time PCR)
  - Digital PCR (dPCR)
  - Droplet digital PCR (ddPCR)
  - CRISPR
  - Other:
19 What type of statistical approaches did you use to analyze your results (select all that apply)?
  - Graphical analyses
  - Descriptive statistics
  - Generalized linear models
  - Site occupancy model
  - Other:

*Dissemination of findings + results*

20 How did you communicate your findings with others (select all that apply)?
  - Professional conferences
  - Direct communication with state/federal managers, researchers
  - Direct communication with user groups (e.g., subsistence, commercial, recreational harvesters)
  - Public meetings or outreach events
  - Peer‐reviewed article
  - Results have not been communicated to those outside of the core research group
  - Other:

*Barriers in eDNA research*

21 What kinds of barriers have you faced when conducting eDNA research?
  - Insufficient funding
  - Lack of agency/organization support
  - Lack of laboratory access/lack of funding for sample analyses
  - Uncertainty about how to analyze data
  - None. I am well‐versed in eDNA methods and applications.
  - Other:
22 Did you hire anyone outside of your agency to complete any of the following steps (select all that apply)?
  - Field sample collection
  - DNA extractions
  - DNA quantification
  - Metabarcoding
  - Sequencing efforts
  - Computational analyses (i.e., bioinformatics)
  - We did not hire anyone outside of our agency
  - Other:
